# Inhibitory Effectiveness of Gomisin A, a Dibenzocyclooctadiene Lignan Isolated from *Schizandra chinensis*, on the Amplitude and Gating of Voltage-Gated Na^+^ Current

**DOI:** 10.3390/ijms21228816

**Published:** 2020-11-21

**Authors:** Wei-Ting Chang, Sheng-Nan Wu

**Affiliations:** 1 Institute of Clinical Medicine, College of Medicine, National Cheng Kung University, Tainan 70101, Taiwan; cmcvecho2@gmail.com; 2 Chi-Mei Medical Center, Division of Cardiovascular Medicine, Tainan 71004, Taiwan; 3 Department of Biotechnology, Southern Taiwan University of Science and Technology, Tainan 71004, Taiwan; 4 Department of Physiology, National Cheng Kung University Medical College, No. 1, University Road, Tainan 70101, Taiwan; 5 Institute of Basic Medical Sciences, National Cheng Kung University Medical College, Tainan 70101, Taiwan; 6 Department of Medical Research, China Medical University Hospital, China Medical University, Taichung 709, Taiwan

**Keywords:** Gomisin A, voltage-gated Na^+^ current, *erg*-mediated K^+^ current, current kinetics, action current, excitable cell

## Abstract

Gomisin A (Gom A), a lignan isolated from *Schisandra chinensis*, has been reported produce numerous biological activities. However, its action on the ionic mechanisms remains largely unanswered. The present experiments were undertaken to investigate the possible perturbations of Gom A or other related compounds on different types of membrane ionic currents in electrically excitable cells (i.e., pituitary GH_3_ and pancreatic INS-1 cells). The exposure to Gom A led to the differential inhibition of peak and end-pulse components of voltage-gated Na^+^ current (*I*_Na_) in GH_3_ cells with effective IC_50_ of 6.2 and 0.73 μM, respectively. The steady-state inactivation curve of *I*_Na_ in the presence of Gom A was shifted towards a more hyperpolarized potential. However, neither changes in the overall current-voltage relationship nor those for the gating charge of the current were demonstrated. The application of neither morin (10 μM) nor hesperidin (10 μM) perturbed the strength of *I*_Na_, while sesamine could suppress it. However, in the continued presence of Gom A, the addition of sesamine failed to suppress *I*_Na_ further. Gom A also effectively suppressed the strength of persistent *I*_Na_ activated by long ramp voltage command, and further application of tefluthrin effectively attenuated Gom A-mediated inhibition of the current. The presence of Gom A mildly inhibited erg-mediated K^+^ current, while a lack of change in the amplitude of hyperpolarization-activated cation current was observed in its presence. Under cell-attached current recordings, the exposure to Gom A resulted in the decreased firing of spontaneous action currents with a minimal change in AC amplitude. In pancreatic INS-1 cells, the presence of Gom A was also noticed to inhibit peak and end-pulse components of *I*_Na_ differentially with the IC_50_ of 5.9 and 0.84 μM, respectively. Taken together, the emerging results presented herein provide the evidence that Gom A can differentially inhibit peak and sustained *I*_Na_ in endocrine cells (e.g., GH_3_ and INS-1 cells).

## 1. Introduction

Gomisin A (Gom A, wuweizichu B, wǔwèizi chún yĭ), a dietary dibenzocyclooctadiene lignan compound isolated from the hexane of *Schisandra chinensis* fruit extract [1,2,3,4,5,6], has been increasingly demonstrated to have anti-inflammatory, anti-oxidative, antihypertensive, neuroprotective, and anti-proliferative properties [7,8,9,10,11]. For example, this compound was reported to induce protective effects either against hepatic and renal injury induced by CCl_4_ exposure, or nitropropionic acid-induced striatal toxicity, through differential regulation of the MAPK signal transduction pathway [12,13]. It could inhibit COX-2, iNOS, IL-6, TNF-α, and NO through the downregulation of RIP2 and NF-κB activation in mouse macrophages [7,14,15]. Earlier reports have also shown that Gom A was able to exert anti-oxidative effects in osteoblast differentiation and in vascular endothelial cells [11,16], and to induce apoptotic changes in colon carcinoma HCT-116 cells [17]. Alternatively, the active components of *S. chinensis* were recently demonstrated to be detected after the intragastic administration of the lignans in rats [6].

Earlier studies have revealed that Gom A could suppress the contractility of penile corpus cavernosum smooth muscle and that this effect appears to be mediated from the NO-cyclic guanosine monophosphate pathway [18]. It has been also demonstrated that schisandrin, one of the major lignans isolated from *Schisandra chinensis*, could suppress spontaneous contraction in rat colon [19]. Additionally, the ability of crude *Schisandra chinensis* extracts to perturb the function of hypothalamic-pituitary-adrenal and gonadal axis in stressed rats has been previously reported [20,21]. The extracts of *S. chinensis* or *arisanensis* have been noticed to improve either amyloid-β- and scopolamine-induced cognitive impairment or pancreatic β-cell function [9,10,22,23,24,25]. Of particular interest, an earlier study demonstrated the effectiveness of crude *S. chinensis* extract in decreasing prolactin production in pituitary GH_3_ cells, suggesting that such an extract could be therapeutically beneficial for patients with hyperprolactinemia and prolactinoma [26].

Nine isoforms (Na_V_1.1–1.9) are present in mammalian excitable tissues, such as endocrine system [27,28]. Of notice, several inhibitors known to preferentially block the late component of voltage-gated Na^+^ current (*I*_Na_), such as ranolazine and perampanel [29,30]. In particular, ranolazine has been demonstrated to exert the inhibitory action on the brain Na_V_ channels for which mutations are associated with epilepsy; moreover, the isoforms of these channels studied are Na_V_1.1, Na_V_1.2, Na_V_1.3 and Na_V_1.6. Those are typically studied in heterologous expression in mammalian cells and in some cases with transgenic mice [31]. Effect of *S. chinensis* extracts (i.e., schizandrol A) on anti-epileptic activity has been reported [32,33]. However, how Gom A or other related compounds is capable of interacting with Na_V_ channels to alter the strength and/or gating of *I*_Na_ in different types of electrically excitable cells largely remains elusive, although previous reports have demonstrated the ability of gomisin A or *S. chinensis* extracts to perturb the strength of transient receptor potential vanilloid type1 [15,34].

In light of the above-mentioned statements, the objective of this work was to investigate whether the presence of Gom A could exert any modifications on the amplitude or gating of *I*_Na_ identified from pituitary tumor (GH_3_) and pancreatic INS-1 cells. The present observations provide the evidence to show, first and foremost, that cell exposure to Gom can effectively and differentially decrease the magnitude of peak and end-pulse *I*_Na_, in combination with an accentuation in the rate of current inactivation. Under cell-attached current recordings, its presence was also noticed to suppress the firing of spontaneous action currents (ACs) recorded from GH_3_ cells. The reduction of AC firing caused by Gom A could be largely explained by its perturbations on the amplitude and/or gating of INa activated in response to rapid depolarization. Therefore, findings from this study provide novel insights into electrophysiological and pharmacological properties of Gom A or other related compounds (e.g., sesamine).

## 2. Results

### 2.1. Inhibitory Effect of Gom A on Voltage-Gated Na^+^ Current (I_*Na*_) in Pituitary GH_3_ Cells

**Figure 1:**
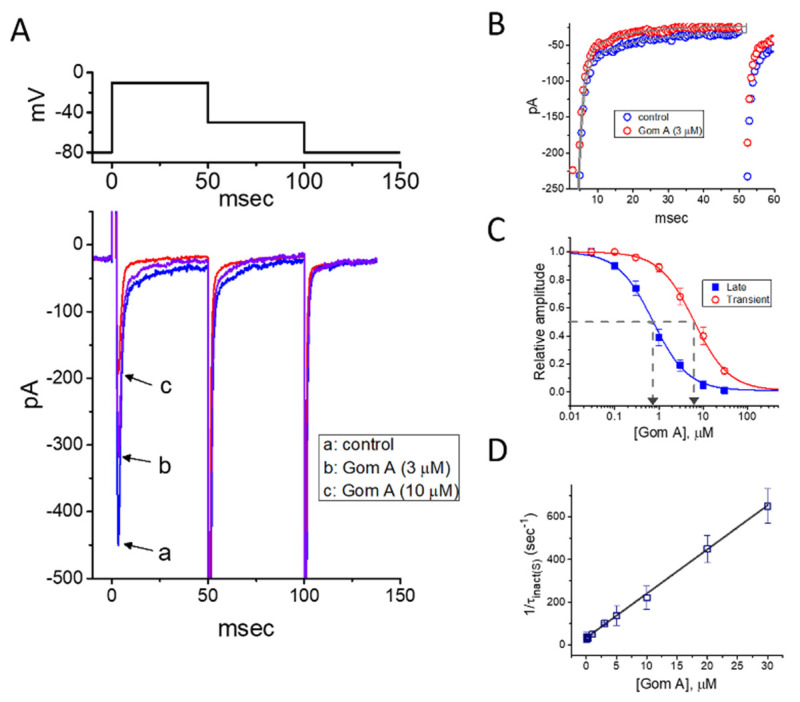
Inhibitory effect of Gom A on voltage-gated Na^+^ current (*I*_Na_) identified in pituitary tumor (GH_3_) cells. In these experiments, we bathed cells in Ca^2+^-free Tyrode’s solution which contained 10 mM tetraethylammonium chloride, and the pipette used was backfilled with Cs^+^-containing solution. (**A**) Representative *I*_Na_ traces in response to membrane depolarization (indicated in the upper part). Current labeled a is control (i.e., Gom A was not present), and that labeled b or c was taken in the presence of 3 or 10 μM Gom A. (**B**) Time course of *I*_Na_ inactivation trajectory activated by brief depolarization. ○: control; ○: in the presence of 3 μM Gom A. The data point in each trace was reduced by a factor of 5 for better illustration. The continuous line indicated in the gray line was least-squares fit to two exponentials. The time constant in the slow component of current inactivation obtained without or with addition of 3 μM Gom A was estimated to be 23.2 or 11.2 msec, respectively. (**C**) Concentration-dependent inhibitory effect of Gom A on the peak (○) or end-pulse (■) component of *I*_Na_ activated by brief depolarizing pulse (mean ± SEM; *n* = 9). Current amplitudes at different concentrations of Gom A were taken at the beginning of the depolarizing pulse from −80 to −10 mV. The IC_50_ value required for Gom A-mediated inhibition of peak or end-pulse *I*_Na_ present in these cells (as indicated in arrow of the dashed vertical line) was calculated to be 6.2 or 0.73 μM, respectively. (**D**) The relation of the reciprocal of τ_inact(S)_ (i.e., slow component of current inactivation) versus the Gom A concentration (mean ± SEM; *n* = 8). The value of current inactivation was measured as the examined cell was rapidly depolarizing from −80 to −10 mV with a duration of 50 msec. According to minimal reaction scheme stated under “Materials and Methods”, forward (*k*_+1_*) or backward (*k*_−1_) rate constants, determined by the slope or the *y*-axis intercept of the interpolated line, was yielded to be·20.7 s^−1^·μM^−1^ or 34.0·s^−1^, respectively.

In the first stage of experiments, we evaluated the possible modifications on *I*_Na_ caused by Gom A in these cells. We bathed cells in Ca^2+^-free Tyrode’s solution, where Ca^2+^ currents or Ca^2+^-activated K^+^ currents are mostly diminished, and the recording electrode was backfilled with Cs^+^-containing solution. As the whole-cell current recordings became established (i.e., membrane patch under the electrode was abruptly broken by suction), the examined cells were clamped at the level of −80 mV, and an abrupt 50-msec membrane depolarization to −10 mV followed by return to −50 mV was thereafter applied (Figure 1). Under this experimental profile, an inward current activated in response to brief depolarizing step was evoked, and it was regarded as an *I*_Na_ [35]. After 1 min of continuous exposure of cells to Gom A, a concentration-dependent drop in the amplitude of both peak and end-pulse components of *I*_Na_ was noticed, concurrently with an evident increase in the inactivation and deactivation rate of the current (Figure 1A,B), despite a lack of change in the activation rate of the current was demonstrated in the presence of Gom A. For example, the addition of 3 μM Gom A resulted in a reduction of current amplitude at the start of depolarizing voltage command from 447 ± 28 to 318 ± 21 pA (*n* = 8, *p* < 0.05). After the agent was washed out, the current amplitude returned to 438 ± 25 pA (*n* = 7, *p* < 0.05). Moreover, apart from the inhibition of current magnitude, the value of time constant in the slow component of current inactivation (τ_inact(S)_) taken in response to brief depolarization, was concurrently diminished during the exposure to this agent (Figure 1A,B). For example, the addition of 3 μM Gom A to the bath resulted in a noticeable decrease in the τ_inact(S)_ value to 11.1 ± 0.8 msec (*n* = 8, *p* < 0.05) from a control value of 23.4 ± 1.2 msec (*n* = 8). Meanwhile, in the presence of 3 μM Gom A, the time constant of deactivating *I*_Na_ obtained following return to the level of −50 mV was decreased from 22.9 ± 1.1 to 12.2 ± 0.9 msec (*n* = 8, *p* < 0.05). However, the value for the time constant in neither current activation rate nor the fast component in the inactivation time constant of the current was modified in the presence of 3 μM Gom A. Alternatively, the value of d*I*/d*t* during rapid rising phase of *I*_Na_ remained unchanged in the presence of 3 μM Gom A (256 ± 14 pA/msec; control) versus 257 ± 16 pA msec (in the presence of Gom A), *n* = 8, (*p* > 0.05), suggesting that the activation kinetics was not altered in its presence.

The depressant effect of Gom A with different concentrations on the peak or end-pulse component of *I*_Na_ activated by 50-msec short depolarization was thereafter evaluated. The concentration-dependent relationship of the inhibitory effect of this agent on *I*_Na_ amplitude (i.e., peak and end-pulse *I*_Na_) in GH_3_ cells was derived and is hence illustrated in Figure 1C. According to the modified Hill equation stated under “Materials and Methods”, the resultant concentration-dependent relationships were analyzed with least-squares approximation, the IC_50_ value of Gom A which was needed for the inhibition of transient or late *I*_Na_ was then yielded to be 6.2 or 0.73 μM, respectively. The results enable us to suggest that the exposure to Gom A could differently and effectively decrease the amplitude of transient and late *I*_Na_.

### 2.2. Evaluation of Time-Dependent Attenuation of I_**Na**_ Inactivation Caused by the Presence of Gom A

It was noticeable that increasing Gom A not simply led to decreased amplitude in the peak *I*_Na_, but it produced an evident elevation in the strength of current inactivation as well. On the basis of the first-order reaction scheme elaborated under “Materials and Methods”, the relationship between 1/τ_inact(S)_ and the Gom A concentration was then approximated to be linear with a correlation coefficient of 0.97 (Figure 1D). As the values of both slope and y-intercept were evaluated, the resultant forward (*k*_+1_*) or backward (*k*_−1_) rate constant was calculated to 20.7 s^−1^·μM^−1^ or 34.0·s^−1^, respectively. Owing to these rate constants, the apparent dissociation constant (i.e., *K*_D_ = *k*_−1_/*k*_+1_*) required for the binding of Gom A to Na_V_ channels was consequently yielded to be 1.64 μM. Of interest, this value is closely similar to the estimated IC_50_ value for Gom A-perturbed inhibition of late *I*_Na_ determined from the concentration-response curve described above (Figure 1C).

### 2.3. Mean I-V Relationship and the Steady-State Inactivation Curve of Peak I_*Na*_ Obtained in the Absence or Presence of Gom A

**Figure 2:**
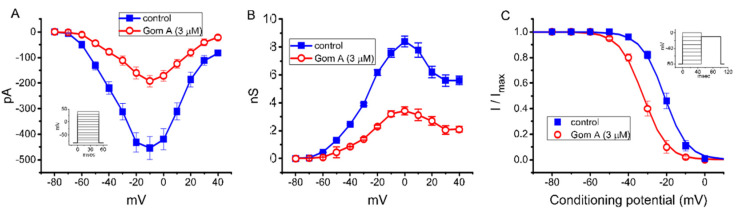
Effect of Gom A on mean current-voltage (*I-V*) relation, conductance versus voltage relation, and the steady-state inactivation curve of *I*_Na_ measured from GH_3_ cells. The experiments were conducted in cells bathed in Ca^2+^-free Tyrode’s solution and we backfilled the solution by using Cs^+^-containing solution. (**A**) Mean *I-V* relation of peak *I*_Na_ in the absence (■) or presence (○) of 3 μM Gom A (mean ± SEM; *n* = 8). Current amplitude was taken at the beginning of depolarizing voltage command. Of notice, the peak amplitude of *I*_Na_ was decreased after the addition of Gom A, with no change the overall *I-V* relation of the current. (**B**) Mean conductance versus voltage relation of peak *I*_Na_ in the control (■) or during the exposure (○) to 3 μM Gom A (mean ± SEM; *n* = 8). (**C**) Steady-state inactivation curve of *I*_Na_ obtained in the control (■) or during the exposure to 3 μM Gom A (mean ± SEM; *n* = 8). The modified Boltzmann equation detailed under “Materials and Methods” was used to fit the experimental data with the goodness-of-fit test. Of notice, the presence of Gom A (3 μM) causes a leftward shift in the inactivation curve of the current by about 11 mV; however, no change in the gating charge of the current was demonstrated in its presence. Inset in (**A**,**C**) indicates the voltage-clamp protocol applied.

As demonstrated in Figure 2A, the effects of Gom A on peak *I*_Na_ were further examined at different membrane potentials, and an *I-V* relationship of the current was established. The observed overall *I-V* relationship of peak *I*_Na_ remained unchanged in the presence of 3 μM Gom A. To characterize the inhibitory effect of Gom A on *I*_Na_, we continued to explore whether there were any adjustments in the steady-state inactivation curve of peak *I*_Na_ during the exposure to this compound. The relationship of conductance versus membrane potential for peak *I*_Na_ taken with or without addition of 3 μM GomA is illustrated in Figure 2B. Figure 2C depicts the steady-state inactivation curve taken without or with addition of 3 μM Gom A. The data with respect to the relationship of conditioning potential versus normalized amplitude of peak *I*_Na_ were established and then goodness-of-fit with a Boltzmann function described under “Materials and Methods”. The resultant value for half-maximal inactivation (*V*_1/2_) or the apparent gating charge (*q*) in the control (i.e., Gom A was not present) was −21.2 ± 1.0 mV or 4.3 ± 0.7 e (*n* = 7), respectively, whereas, during the exposure to 3 μM Gom A, the value of *V*_1/2_ or *q* was −32.2 ± 1.1 mV or 4.4 ± 0.7 e (*n* = 7), respectively. These data therefore enable us to reflect that the steady-state *I*_Na_ inactivation curve in the presence of Gom A was shifted in a leftward direction, with no evident adjustment in the gating charge of the current, and that this agent exerts depressant action on peak *I*_Na_ in a voltage-dependent fashion in these cells.

### 2.4. Comparison of Effects of Morin, Hesperidin, Gom A, Sesamine, Gom A Plus Sesamine on Peak I_*Na*_ Identified in GH_*3*_ Cells

**Figure 3:**
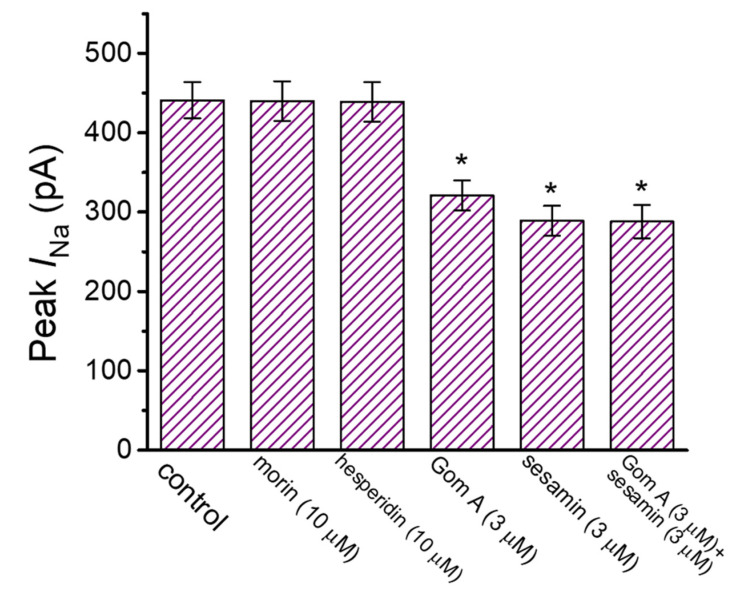
Comparison in effects of morin, hesperidin, Gom A, sesamine, Gom A plus sesamine on the amplitude of peak *I*_Na_ measured from GH_3_ cells (mean ± SEM; *n* = 7–8). The experiments were undertaken in cells bathed in Ca^2+^-free Tyrode’s solution and we filled up the electrode with Cs^+^-containing solution. In an effort to activate *I*_Na_ in these cells, a 50-msec square voltage-clamp pulse to −10 mV from the holding potential of −80 mV was delivered to the examined cell, and current amplitude was measured at the start of depolarizing pulse. * Significantly different from control (*p* < 0.05).

We also further evaluated and compared effects of different compounds (e.g., morin, hesperidin and sesamin) which could be contained in the extracts of *S. chinensis* (Figure 3) [35,36]. The addition of neither morin nor hesperidin was noticed to perturb the amplitude of peak *I*_Na_ activated by rapid membrane depolarization. However, as reported recently [35], when GH_3_-cells were exposed to sesamine (3 μM), the peak amplitude of the current was significantly decreased. Morin and hesperidin contained in *S. chinensis* extracts are the phenolic compounds (i.e., flavonoid). Moreover, in the continued presence of Gom A (3 μM), subsequent application of sesamine (3 μM) failed to decrease the peak amplitude of *I*_Na_ further. It is therefore plausible to anticipate that Gom A and sesamine interact with similar motifs to perturb the strength and/or the gating of *I*_Na_ in these cells.

### 2.5. The Amplitude of Persistent Na^+^ Current (I_*Na*__(__*P*__)_) Decreased by Gom A

**Figure 4:**
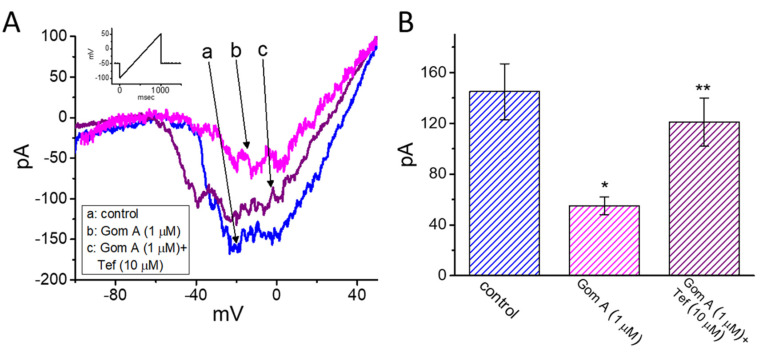
Effects of Gom A and Gom A plus tefluthrin on the persistent component of *I*_Na_ (*I*_Na(P)_) in GH_3_ cells. The whole-cell current recordings were conducted in cells which was activated by a-sec long ramp pulse from −100 to +50 mV. (**A**) Representative current traces obtained in the control (i.e., Gom A was not present, a) and during the exposure to 1 μM Gom A (b) or 1 μM Gom A plus 10 μM tefluthrin (Tef) (b). Inset indicates the voltage protocol applied. (**B**) Summary bar graph showing effects of Gom A and Gom A plus tefluthrin on the amplitude of *I*_Na(P)_ (i.e., tetrodotoxin-sensitive current) (mean ± SEM; *n* = 7). Current amplitude was measured at the level of −10 mV when the examined cell was ramp-pulsed from −100 to +50 mV with a duration of 1 sec. * indicates significantly from control (*p* < 0.05) and ** significantly from 1 μM Gom A alone group (*p* < 0.05).

We continued to explore whether the presence of Gom A could be able to perturb the strength of *I*_Na(P)_ measured from GH_3_ cells. As the whole-cell current recordings were established, we held the cell in voltage clamp at −50 mV and a 1-s ramp pulse from −100 to +50 mV was applied to evoke *I*_Na(P)_ [37]. Of notice, when the cells were exposed to 1 μM Gom A, the amplitude of *I*_Na(P)_ activated under such slow ramp command was diminished (Figure 4A,B), as demonstrated by a conceivable reduction of current amplitude (i.e., measured at the level of −10 mV) from 145 ± 22 to 55 ± 7 pA (*n* = 7, *p* < 0.05). After washout of the agent, *I*_Na(P)_ amplitude at the same level of membrane potential was returned to 141 ± 19 pA (*n* = 7, *p* < 0.05). Moreover, during the continued exposure to 1 μM Gom A, subsequent application of tefluthrin (10 μM) was able to counteract Gom A-attenuated amplitude of *I*_Na(P)_ effectively in GH_3_ cells, as evidenced by the increase of *I*_Na(P)_ amplitude to 121 ± 19 (*n* = 7, *p* < 0.05). Tefluthrin, a type-I pyrethroid insecticide, was previously demonstrated to augment the strength and inactivation time constant of *I*_Na_ [37,38,39]. It is conceivable, therefore, that Gom A effectively suppresses the amplitude of *I*_Na(P)_ in response to the long ramp pulse. Moreover, as cells were continually exposed to Gom A (1 μM), subsequent addition tetrodotoxin (1 μM) effectively decreased *I*_Na(P)_ elicited in response to such ramp pulse to 5 ± 1 pA (*n* = 7, *p* < 0.05). Of notice, the presence of Gom A (1 μM) could decrease *I*_Na(P)_ by 62%, while it diminished the peak or end-pulse component of *I*_Na_ by 11% or 61%, respectively.

### 2.6. Effect of Gom A and Gom A Plus Ranolazine on erg-mediated K^+^ Current (I_*K*(*erg*)_)

**Figure 5:**
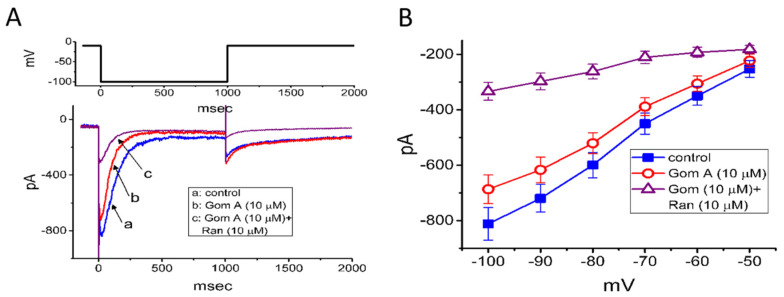
Mild inhibition of erg-mediated K^+^ current caused by Gom A in GH3 cells. In this set of experiments, cells were bathed in high-K^+^, Ca^2+^-free solution containing 1 μM tetrodotoxin and, for current recordings, we backfilled the pipette by using K^+^-containing solution. (**A**) Representative IK(erg) traces obtained in the absence (a) and presence of 10 μM Gom A (b) or 10 μM Gom A plus 10 μM ranolazine (Ran, c). The upper part shows the voltage protocol delivered. (**B**) Mean I-V relationship of IK(erg) taken in the control (■) and during cell exposure to 10 μM Gom A (○) or 10 μM Gom A plus 10 μM ranolazine (mean ± SEM; *n* = 7). Current amplitude was taken at the beginning of each hyperpolarizing step.

We further extended to determine whether Gom A might perturb voltage-gated K^+^ current (e.g., *I*_K(erg)_) in GH_3_ cells. In attempts to amplify the magnitude of *I*_K(erg)_ [29,40], the experiments were conducted in cells bathed in high-K^+^, Ca^2+^-free solution, while the recording pipette was filled with K^+^-containing solution. The compositions of these solutions were detailed under “Materials and Methods”. In these experiments, to evoke *I*_K(erg)_, the examined cell was 1-sec hyperpolarized from −10 to −100 mV. As cells were exposed to Gom A at a concentration of 3 μM, no significant change in *I*_K(erg)_ amplitude was demonstrated (815 ± 61 pA [control] versus 812 ± 64 pA [in the presence of 3 μM Gom A], *n* = 7, *p* > 0.05). However, as depicted in Figure 5A, the addition of 10 μM Gom A was noticed to decrease the amplitude of deactivating *I*_K(erg)_ significantly from 812 ± 59 to 687 ± 52 pA (*n* = 8, *p* < 0.05). After the agent was washed out, current amplitude became returned to 807 ± 58 pA (*n* = 7, *p* < 0.05). Moreover, as cells were continually exposed to 10 μM Gom A, further application of ranolazine (10 μM) resulted in an additional reduction of current amplitude, as demonstrated by the decrease of *I*_K(erg)_ amplitude to 334 ± 32 pA (*n* = 8, *p* < 0.05). Apart from being a blocker of late *I*_Na_, ranolazine has been previously reported to modify the amplitude and gating of *I*_K(erg)_ [29,33]. Alternatively, as cells were continually exposed to 10 μM Gom A, further addition of E-4031 (10 μM), a blocker of *I*_K(erg)_ fully abolished *I*_K(erg)_ amplitude. The mean *I-V* relationships of *I*_K(erg)_ obtained in the control and in the presence of 10 μM Gom A or 10 μM Gom A plus 10 μM ranolazine were constructed and are hence illustrated in Figure 5B. Under our voltage-clamp conditions, the experimental observations led us to suggest that, distinguishable from *I*_Na_, the *I*_K(erg)_ inherently in GH_3_ cells is to some extent relatively resistant to being blocked by Gom A.

### 2.7. Failure of Gom A to Alter Hyperpolarization-Activated Cation Current (I_*h*_) in GH_*3*_ Cells

**Figure 6:**
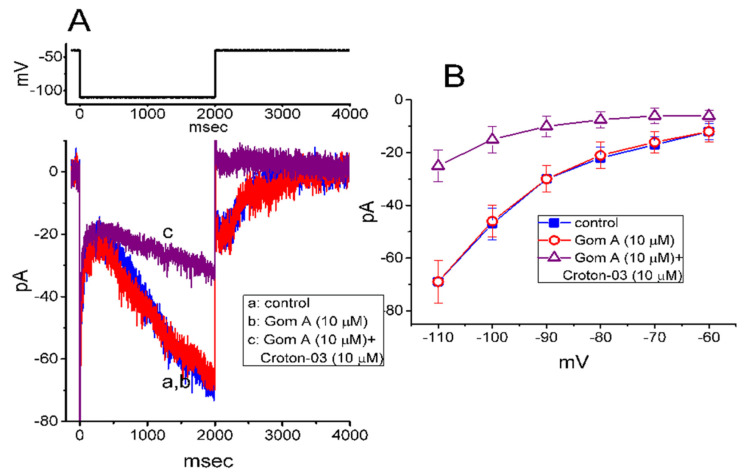
Inability of Gom A to inhibit hyperpolarization-activated cation current (Ih) in GH3 cells. In this set of whole-cell experiments, we bathed cells in Ca^2+^-free Tyrode’s solution containing 1 μM tetrodotoxin and we filled the backfilling solution with K^+^-containing solution. (**A**) Representative Ih traces taken in the absence (a) and presence of 10 μM Gom A (b) or 10 μM Gom A plus 10 μM croton-03 (c). The voltage protocol used is illustrated in the upper part. (**B**) Mean I-V relationship of Ih (i.e., ivabradine-sensitive current) collected in the control (■) and during cell exposure to 10 μM Gom A (○) or 10 μM Gom A plus 10 μM croton-03 (mean ± SEM; *n* = 8). The cell examined was held at −10 mV and a series of hyperpolarizing commands ranging between −110 and −60 mV with a duration of 2 sec were delivered. Current amplitude was taken at the end of each hyperpolarizing step applied.

In a separate set of experiments, we continued to test whether this agent produced any effect on the amplitude and/or gating of *I*_h_ inherently in these cells [35,41]. To measure *I*_h_, cells were bathed in Ca^2+^-free Tyrode’s solution and we filled the electrode by using K^+^-containing solution. As shown in Figure 6A, when the 2-s hyperpolarizing pulse from −40 to −110 mV was applied to the cell, the *I*_h_ with the slowly activating time course was readily evoked [35,41]. However, as cells were exposed to 10 μM Gom A, neither the amplitude nor gating of *I*_h_ were perturbed (69 ± 7 pA [in the control] versus 69 ± 8 pA [in the presence of Gom A], *n* = 8, *p* > 0.05). Moreover, in the continued presence of 10 μM Gom A, subsequent addition of 10 μM croton-03 was able to decrease *I*_h_ amplitude effectively, as demonstrated by a significant reduction of current amplitude to 25 ± 6 pA (*n* = 8, *p* < 0.05). Similarly, as cells were continually exposed to 10 mM Gom A, further application of 10 μM ivabradine, a blocker of *I*_h_, fully abolished *I*_h_ amplitude. Figure 6B illustrates the mean *I-V* relationships of *I*_h_ (measured at the end of each hyperpolarizing step) taken in the control and during the exposure to 10 μM Gom A or 10 μM Gom A plus 10 μM croton-03. As such, consistent with previous reports [42], the present observations showed that croton-03 was effective at inhibiting *I*_h_. However, lack of change in *I*_h_ amplitude or gating was demonstrated in the presence of Gom A alone. Unlike *I*_Na_, the hyperpolarization-activated *I*_h_ identified in these cells is subject to be resistant to the modification by Gom A.

### 2.8. Inhibitory Effect of Gom A on Spontaneous Action Currents (ACs) Identified in GH_*3*_ Cells

**Figure 7:**
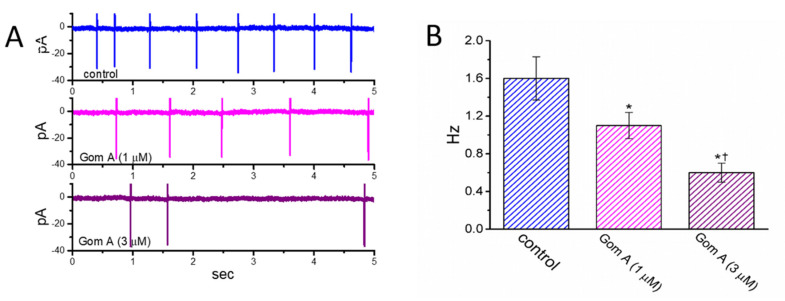
Inhibitory effect of Gom A on the firing of action currents (ACs) recorded from voltage-clamped GH_3_ cells. In this set of cell-attached current recordings, we bathed cells in normal Tyrode’s solution which contained 1.8 mM CaCl_2_, the electrode was filled with K^+^-containing solution, and the potential was maintained at the resting of the cell (~−70 mV). (**A**) Representative AC traces obtained in the control (upper) and during cell exposure to 1 μM Gom A (middle) or 3 μM Gom A (lower). Of notice, the inward deflection in each traces indicates the occurrence of AC. (**B**) Summary bar graph showing inhibitory effect of Gom A (1 and 3 μM) on the firing frequency of spontaneous ACs identified from these cells (mean ± SEM; *n* = 8). * indicates significantly different from control (*p* < 0.05) and ^†^ significantly different from Gom A (1 μM) alone group (*p* < 0.05).

We then continued to examine whether Gom A had any effects on AC firing in these cells. In these cell-attached current recordings, we immersed cells in normal Tyrode’s solution containing 1.8 mM CaCl_2_, and we then voltage-clamped the examined cell at the resting potential. As demonstrated in Figure 7, the addition of different concentrations (1 and 3 μM) of Gom A diminished the AC frequency significantly. However, no change in AC amplitude was noticed in its presence. For example, as cell cells were exposed to Gom A (3 μM), the firing frequency of ACs detected in GH_3_ cells was decreased to 0.6 ± 0.1 Hz (*n* = 7, *p* < 0.05) from a control value of 1.6 ± 0.2 Hz (*n* = 7). After this agent was washed out, the firing frequency was returned to 1.5 ± 0.2 Hz (*n* = 7, *p* < 0.05). It is conceivable, therefore, that the Gom A-perturbed reduction of AC firing in these cells is closely linked to its inhibition of *I*_Na_ described above.

### 2.9. Effect of Gom A on I_*Na*_ in INS-1 Cells

**Figure 8:**
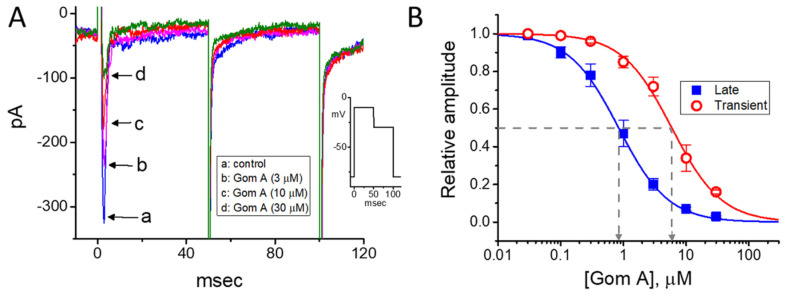
Inhibitory effect of Gom A on *I*_Na_ measured from pancreatic INS-1 cells. Cells were bathed in Ca^2+^-free Tyrode’s solution containing CdCl_2_ (0.5 mM). (**A**) Representative *I*_Na_ traces obtained in the absence (a) and presence of 3 μM Gom A (b), 10 μM Gom A (c), or 30 μM Gom A (d). As indicated in inset of (**A**), in these experiments, the hold potential was set at −80 and the 50-msec depolarlzing pulse to −10 mV followed by a return to the level of −30 mV with a duration of 50 msec. (**B**) Concentration-dependent inhibition of peak (■) or end-pulse (○) component of *I*_Na_ by Gom A. Current amplitude during the exposure to different concentrations of Gom A was taken at the beginning of depolarizing pulse from −80 to −10 mV. By use of least-squares non-linear regression approximation, the IC_50_ value required for Gom A-mediated inhibition of transient or late *I*_Na_ observed in these cells (as indicated in arrow of the dashed vertical line) was yielded to be 5.9 or 0.84 μM, respectively.

The extracts of *S. chinensis* or *arisanensis* have been previously demonstrated to modify pancreatic β-cell function [23,25,43]. Ranolazine, known to be an inhibitor of late *I*_Na_, has been previously noticed to influence glycemic control [44]. Therefore, in a final set of experiments, effects of Gom A on another type of electrically excitable endocrine cells (i.e., INS-1 cells) were further evaluated. INS-1 cells were bathed Ca^2+^-free Tyrode’s solution and we filled up the recording pipette by using Cs^+^-containing solution. As depicted in Figure 8, as INS-1 cells were exposed to different concentrations (0.03–30 μM) of Gom A, the amplitude of *I*_Na_ was progressively decreased. The effective IC_50_ value of Gom A-perturbed inhibition of peak or end-pulse component of *I*_Na_ was estimated to be 5.9 or 0.84 μM, respectively. Findings from these experimental data are in keeping with those elaborated above in GH_3_ cells.

## 3. Discussion

The present results demonstrated that the presence of Gom A was able to produce an inhibitory action on *I*_Na_ in GH_3_ cells in a concentration-, a time- and state-dependent manner. Cell exposure to Gom A was also noticed to accentuate the rate of *I*_Na_ inactivation, particularly at the slow component of current inactivation. The inhibitory action on *I*_Na_ tended to be rapidly developing and readily washed out, and it would concurrently correlate in time with a significant raise in the inactivation rate of the current activated by short depolarizing pulse, while the activation kinetics of *I*_Na_ remained unchanged in the presence of Gom A. The observed effect of Gom A on the strength and gating *I*_Na_ primarily ascribed from this molecule acting largely on the Na_V_ channel itself or its accessory subunits.

In pituitary GH_3_ cells, Gom A, known to be a lignan isolated from *S. chinensis*, differentially and effectively inhibited the transient or late components of *I*_Na_ with effective IC_50_ value of 6.2 or 0.73 μM. respectively. According to minimal binding scheme, the approximated K_D_ value for Gom A-perturbed raise in current inactivation was also calculated to be 1.64 μM, a value that tends to be similar to that needed to lessen the magnitude of late component of *I*_Na_. It is therefore possible that the Gom A molecule preferentially binds to and interacts with the open state or conformation of the Na_V_ channel, hence producing a conceivable reduction in both amplitude and inactivation time constant of *I*_Na_. The presence of this agent was also noticed to inhibit the amplitude of *I*_Na(P)_ activated by a long ramp pulse, and its inhibition of *I*_Na(P)_ was able to be attenuated by subsequent addition of tefluthrin, an activator of *I*_Na_ [37,39]. Therefore, the Gom A-mediated reduction in *I*_Na(P)_ could also be explained by the accelerated deactivation of Na_V_ channel, and the possibility that Gom A might bind preferentially to the inactivated state of the channel could not be excluded. Under our cell-attached current recordings, the observed reduction of AC firing caused by Gom A could be largely explained by its effectiveness in the inhibition of peak and end-pulse *I*_Na_ measured from GH_3_ cells. However, further studies are still needed to evaluate the extent to which this compound has therapeutic relevance in the treatments of patients with hyperprolactemia or prolactinoma as demonstrated previously [26].

In our study, the addition of Gom A was not only able to decrease the maximal conductance of peak *I*_Na_ but caused a negative (leftward) shift in the steady-state inactivation curve of the current as well. The lack of any change in the gating charge of the inactivation curve during the exposure to this agent thus enables us to indicate that the interaction with the Na_V_ channel is not mediated by a direct effect on the voltage sensor per se and that its binding site is likely to lie outside of the transmembrane field of the channel. An important consequence of a leftward shift of the inactivation curve is the decreased membrane excitability at voltages near the resting potential, accompanied by lessened AC firing identified in GH_3_ cells. Under this scenario, the sensitivity of Gom A in non-voltage-clamped neurons or endocrine cells would rely on various confounding factors that include the preexisting level of resting potential, the firing of spontaneous action potentials, and the concentration of Gom A applied.

Gom A was recently demonstrated to penetrate into brain tissue [6,45]. Moreover, the plasma concentration of Gom A measured after oral administration in rats was previously reported to reach around 17 μM [46], a value that is noticeably higher than the IC_50_ values for Gom A-mediated inhibition of both peak and end-pulse *I*_Na_ measured from GH_3_ and INS-1 cells. Therefore, our experimental observations demonstrating the effectiveness of Gom A in modifying membrane ionic currents, which tend to be upstream of its anti-oxidative properties [7,11,14,15], would enable us to indicate that these actions could be one of the underlying ionic mechanisms responsible for its perturbations on membrane excitability in different types of electrically excitable cells (e.g., GH_3_ and INS-1 cells).

In this study, the presence of neither morin nor hesperidin was found to have any changes on the peak *I*_Na_, while sesamine, another lignan, effectively inhibited the strength of the current as disclosed previously [35]. Therefore, Gom A or sesamine, which could be the important constituents contained in *S. chinensis* extracts, was capable of participating in the depressant action on *I*_Na_. However, the inability of subsequent application of sesamine to perturb Gom-A-mediated inhibition of peak *I*_Na_ further, led us to imply that these two compounds tend to bind to similar motifs to interact with the Na_V_ channel. Of notice, according to modeled Na_V_ (i.e., SCN8A-encoded or Na_V_1.6) channels originally developed from a Markovian process (i.e., modified Pan-Cummins model) [47], it seems plausible to anticipate, therefore, that the “Oon”, which represents the on rate of normal inactivation from the open state of the channel, could be a noticeable variable with which Gom A and/or sesamine could interact [35].

Previous work has also unraveled the ability of ranolazine, an inhibitor of late *I*_Na_, to suppress *I*_K(erg)_ amplitude effectively [29,48,49]. However, in this study, Gom A at a concentration of 10 μM only mildly inhibited the amplitude of deactivating *I*_K(erg)_ responding to long-lasting hyperpolarization. Further application of ranolazine (10 μM) or E-4031 (10 μM), still in the continued presence of Gom A, was effective at decreasing *I*_K(erg)_ further. Additionally, there appears to be void of modifications in the strength and gating of *I*_h_ in the presence of Gom A. Therefore, by comparison with the ranolazine action, the inhibitory effect of Gom A on *I*_Na_ identified from GH_3_ or INS-1 cells could be to some extent more specific.

The binding scheme presented in this study enables us to suggest the recovery of Na_V_ channels from the open state. However, of notice, Na_V_-channel isoforms might differ very specifically for this transition. Most isoforms are able to recover from the inactivated to the closed (or resting) state, although open-state recovery observed in a few isoforms (e.g., SCN8A-encoded or Na_V_1.6 current). The Na_V_ isoforms in these cell lines (i.e., GH_3_ and INS-1 cells) have not been identified and this minimal scheme could therefore make an assumption that may not be valid. Additionally, the *I*_Na(P)_ itself is not an indicator of open-state recovery. Taken together, the scheme demonstrated herein does not account for the presence of the closed-state inactivation. As such, it would be interesting to evaluate whether the Na_V_ isoforms in the cells studied are able to transit to the inactivated state without opening activated in response to weak depolarization. In muscle and neuronal Na_V_ channels this state transition is the dominant parameter for steady-state inactivation and is a target of channelopathy-mediated gating defects [50]. It also remains to be further studied with respect to the extent what Gom A or other structurally similar compounds could interact with different isoforms of Na_V_ channels to modify the strength and kinetics of *I*_Na_.

Our study was also extended to demonstrate that, in INS-1 cells, similar to the ranolazine on *I*_Na_ [49], the presence of Gom A was efficacious at inhibiting *I*_Na_ activated by rapid depolarization. The IC_50_ value for its inhibition of peak or end-pulse *I*_Na_ in these cells was yielded to be 5.9 or 0.84 μM, respectively. The experimental observations, therefore, enable us to anticipate that Gom A-mediated inhibition of *I*_Na_ observed in pancreatic β-cells could be closely linked to its perturbations on electrical behavior or other functional activities in various types of electrically excitable cells [18,19,26]. The inhibitory actions presented herein allowed us to provide important insights into the underlying ionic mechanism for Gom A actions [4,8,26].

## 4. Materials and Methods

### 4.1. Chemicals, Drugs and Solutions Used in This Study

Gomisin A (Gom A, wǔwèizi chún yĭ, CFN98990, besigomisin, schisandrin B, schisandrol B, schisantherinol B, schizandrol B, TJN-101; wuwizi alcohol B, wuweizichun B, (6S,7S,13aR)-5,6,7,8-tetrahydro-1,2,3,13-tetramethoxy-6,7-dimethyl-benzo[3,4]cycloocta[1,2-f][1,3]benzodioxol-6-ol, C23H28O7, PubChem CID: CID-3001662), heresperin, and morin were acquired from ChemFaces (Rainbow Biotechnology, Taipei, Taiwan), and E-4031, ivabradine, β-mercaptoethanol, sodium pyruvate, tetrodotoxin, and trypan blue were from Sigma-Aldrich (Merck, Taipei, Taiwan), while ranolazine was from Tocris (Union Biomed, Taipei, Taiwan). Croton-03 or sesamine was kindly provided by Dr. Ping-Chung Kuo (School of Pharmacy, National Cheng Kung University Medical College, Tainan, Taiwan). Unless stated otherwise, culture media (e.g., Ham’s F12 and RPMI-1460 media), fetal bovine or calf serum, horse serum, L-glutamine, and trypsin/EDTA were acquired from HyCloneTM (Thermo Fisher, Level Biotech, Tainan, Taiwan), while other chemicals or reagents, such as CsOH, CsCl_2_, CdCl_2_, EGTA and HEPES, were of analytical grade.

The HEPES-buffered normal Tyrode’s solution used in this study had a composition, which comprised (in mM): NaCl 136.5, KCl 5.4, CaCl_2_ 1.8, MgCl_2_ 0.53, glucose 5.5, and HEPES 5.5 adjusted to pH 7.4 with NaOH. For the measurement of *I*_Na_ or *I*_h_, we bathed cells in Ca^2+^-free Tyrode’s solution in order to minimize the contamination of Ca^2+^-activated K^+^ currents and voltage-gated Ca^2+^ currents. To record *I*_K(erg)_, cells were bathed in high-K^+^, Ca^2+^-free solution which contained (in mM): KCl 130, NaCl 10, MgCl_2_ 3, and HEPES 5 adjusted to pH 7.4 with KOH. For the investigations on *I*_K(erg)_, *I*_h_ or spontaneous action currents (ACs), we backfilled the electrode with the internal solution (in mM): K-aspartate 130, KCl 20, KH_2_PO_4_ 1, MgCl_2_ 1, EGTA 0.1, Na_2_ATP 3, Na_2_GTP 0.1, and HEPES 5 adjusted to pH 7.2 with KOH, while to measure *I*_Na_ and to minimize contamination of K^+^ currents, K^+^ ions in the backfilling solution were substituted for Cs^+^ ions, and pH value was titrated to pH 7.2 with CsOH. All solutions were prepared using demineralized water from a Milli-Q water purification system (Merck, Ltd., Taipei, Taiwan). On the day of experiments, we filtered the bathing or backfilling solution and culture medium by using an Acrodisc^®^ syringe filter with a 0.2-μm Supor^®^ membrane (Bio-Check; New Taipei City, Taiwan).

### 4.2. Cell Preparations

GH_3_, originally acquired from the Bioresources Collection and Research Center ([BCRC-60015]; Hsinchu, Taiwan), were grown in Ham’s F-12 medium supplemented with 15% (by volume) horse serum, 2.5% (by volume) fetal calf serum and 2 mM l-glutamine. The rat INS-1 cell line (clone 832/13) was kindly provided by Dr. Christopher B. Newgard, Duke University, Durham, NC, USA. INS-1 cells were cultured in RPMI-1460 medium with 11.1 mM D-glucose which was supplemented with 10% (by volume) fetal bovine serum, 10 mM HEPES. 2 mM l-glutamine, 1 mM sodium pyruvate, and 50 μM β-mercaptoethanol [35,42,51]. GH_3_ or INS-1 cells were maintained at 37 °C in a 95% air and 5% CO_2_ humidified atmosphere. The viability of these cells was quantified by the trypan blue dye-exclusion method. The electrophysiological recordings were undertaken five or six days after cells had been cultured up to 60–80% confluence.

### 4.3. Electrophysiological Measurements

Prior to measurements, GH_3_ or INS-1 cells were harvested with 1% trypsin/EDTA solution and an aliquot of cell suspension was rapidly transferred to a custom-built recording chamber which was firmly mounted on the stage of an inverted DM-IL microscope (Leica; Major Products, New Taipei City, Taiwan). Cells were bathed at room temperature (20–25 °C) in HEPES-buffered normal Tyrode’s solution, the composition of which was detailed above. To prepare patch electrodes from Kimax-51 capillaries (outer diameter of 1.5 to 1.8 mm) (#34500-99; Kimble, Dogger. New Taipei City, Taiwan), we used either a horizontal P-97 Flaming/Brown puller (Sutter; Taiwan Instrument, Tainan, Taiwan) or a vertical PP-830 puller (Narishige; Taiwan Instrument, Tainan, Taiwan), and we then further fire-polished the tip with MF-83 microforge (Narishige). During the recordings, the electrodes, which had the resistance ranging between 3 and 5 MΩ when filled with different internal solution stated above, were tightly mounted to the holder, and they were then maneuvered by using MX-4 manipulator (Narishige) and finely operated by MHW-3 hydraulic micromanipulator (Narishige). We performed the patch-clamp technique with dynamic adaptive suctioning (i.e., decremental change of suction pressure in response to a progressive increase in the electrode resistance) in whole-cell or cell-attached configuration by an Axopatch-200B (Molecular Devices; Advance Biotech, New Taipei City, Taiwan) or RK-400 (Bio-Logic, Claix, France) amplifier [41,52,53]. Liquid junction potentials, which commonly developed at pipette tip when the composition of internal solution was different from that in the bath, were nulled shortly before seal formation was made, and whole-cell data were corrected by such junction potentials.

Action currents (ACs), which corresponds with the occurrence of action potentials detected under whole-cell voltage measurements, were undertaken in cell-attached current recordings [40,53,54]. For AC measurements, the holding voltage was set at the level of the resting potential (~−70 mV). As such, AC measurements were achieved to ensure the quantification of the underlying firing frequency with being void of clear changes in intracellular milieu. The derails of data recordings and analyses were listed in the Supplementary Materials.

### 4.4. Statistical Analyses

Linearized or non-linearized curve-fitting procedure (i.e., least-squares approximation) used in this study was undertaken by using either the SOLVER function (i.e., the generalized reduced gradient method of iteration) embedded in Excel 2016 (Microsoft) or OriginPro 2016 (OriginLab). The experimental results are presented as mean value ± standard error of the mean (SEM) with sample sizes (*n*) indicative of the cell numbers from which the data were collected. Student’s *t*-test (paired or unpaired) was initially employed for the statistical analyses. As the statistical difference among different groups was necessarily determined, we performed either analysis of variance (ANOVA)-1 or ANOVA-2 with or without repeated measures followed by Duncan’s post hoc test. A *p*-value of < 0.05 was considered to indicate statistical difference.

## 5. Conclusions

Collectively, our findings showed that Gom A can differentially inhibit peak and sustained *I*_Na_ in endocrine cells. This would provide important insights into the underlying ionic mechanism for Gom A actions.

## Supplementary Materials

The following are available online at https://www.mdpi.com/1422-0067/21/22/8816/s1.

## Abbreviations

AC	action current
ANOVA	analysis of variance
Gom A	gomisin A
*I*_h_	hyperpolarization-activated cation current
*I*_K(erg)_	*erg*-mediated K^+^ current
*I*_Na_	voltage-gated Na^+^ current
*I*_Na(P)_	persistent Na^+^ current
*K*_D_	dissociation constant;
Na_V_ channel	voltage-gated Na^+^ channel
t_inact(S)_	time constant for slow component of *I*_Na_ inactivation.
