# Supplementary material for: Inhibitory Effectiveness of Gomisin A, a Dibenzocyclooctadiene Lignan Isolated from Schizandra chinensis, on the Amplitude and Gating of Voltage-Gated Na+ Current
Source: Int J Mol Sci. 2020 Nov 21;21(22):8816. doi: 10.3390/ijms21228816 (PMC7700137; doi:10.3390/ijms21228816)
Supplement: Supplementary file 1 [file ijms-21-08816-s001.pdf]

Data recordings

The signals encompassing voltage and current tracings were displayed and online stored in an ASUS VivoBook Flip 14 laptop computer (ASUS, Tainan, Taiwan) at 10 kHz equipped with Digidata-1440A interface (Molecular Devices), which was controlled by pCLAMP 10.7 software (Molecular Devices). Current signals were low-pass filtered at 2 KHz with a FL-4 four-pole Bessel filter (Dagan; Advance Biotech, Taipei, Taiwan). In some separate set of experiments, the data were digitally acquired by PowerLab system with LabChart 7.0 software (AD Instruments; KYS Technology, Taipei, Taiwan). The signals collected during the measurements were offline analyzed by using either pCLAMP 10.7 (Molecular Devices), OriginPro 2016 (OriginLab, Northampton, MA), or custom-made macros built in Excel 2016 spreadsheet running on Windows-10 (Microsoft, Redmond, VA). In attempts to evaluate the current-voltage (I-V) relationships and the steady-state inactivation curve for ion current (e.g., I_{Na}), a family of rectangular or ramp voltage commands created from pCLAMP 10-7 were designed and then applied through digital-to-analog conversion. When high-frequency stimuli were required, an Astro-Med Grass S88X dual output pulse stimulator (Grass Technologies, West Warwick, RI) would be used.

Data analyses

In attempts to evaluate concentration-dependent inhibition of Gom A on the

peak or end-pulse amplitude of I_{Na} activated by rapid depolarizing pulse, we immersed cells in Ca^{2+}-free Tyrode's solution. The peak or end-pulse component of I_{Na} activated by the depolarizing voltage command from -80 to -10 mV was measured at the start or end of the pulse, respectively. The Gom A concentration required to suppress 50% of current amplitude (i.e., peak or end-pulse I_{Na}) was calculated according to the modified Hill equation:

$$\text{Relative amplitude} = \frac{[\text{Gom A}]^{-\text{n}_\text{H}} \times (1-a)}{[\text{Gom A}]^{-\text{n}_\text{H}} + \text{IC}_{50}^{-\text{n}_\text{H}}} + a$$

where IC_{50} or n_H denotes the concentration of Gom A required for a 50% inhibition of current amplitude or the Hill coefficient, respectively, and maximal inhibition (i.e., 1-*a*) was also estimated from this equation.

The inhibitory effect of Gom A on I_{Na} can be explained by a state-dependent manner, since this compound preferentially binds to the open and/or inactivated state of Na_V channels. A minimal binding scheme for its binding to the channel was given by:

$$\text{Closed} \underset{\beta}{\overset{\alpha}{\rightleftharpoons}} \text{Open} \underset{k_{-1}}{\overset{k_{+1}^{*}\cdot[\text{Gom A}]}{\rightleftharpoons}} \text{Inactivated}$$

were α or β is the voltage-dependent rate constant for the opening or closing of Na_V channels, respectively; k_{+1}^{*} or k_{-1} is that for forward (or on) or backward (or off) rate of current inactivation, respectively; and [Gom A] is the gomisin A (i.e., [Gom A]) concentration applied. Closed, open, or inactivated shown above represents either

closed (or resting), open, or inactivated state of the Na_V channel, respectively.

Forward or backward rate constant, k_{+1}^* or k_{-1}, was further determined from the time constant ($\tau_{inact(S)}$) in the slow component of I_{Na} inactivation activated by the depolarizing voltage-clamp command from -80 to -10 mV. The $\tau_{inact(S)}$ values collected with or without addition of different Gom A concentrations became evaluated by fitting inactivation trajectory of each current trace with double exponential curve (i.e., the fast and slow components of current inactivation). The rate constants were then estimated by using a first-order kinetic scheme:

$$\frac{1}{\tau_{\mathrm{inact(S)}}} = {k_{+1}}^* \times [\mathrm{Gom\ A}] + k_{-1}$$

where k_{+1}^* or k_{-1} was, respectively, obtained from the regression slope or the y-intercept (i.e., the value of y when [Gom A] = 0) of the linear regression. The regression line was derived by interpolating the reciprocal time constants (i.e., $1/\tau_{inact(S)}$) versus different Gom A concentrations (0.03-30 μM). Correspondingly, the plot of $\tau_{inact(S)}$ versus Gom A was constructed and the dissociation constant (K_D, k-1/k_{+1}^*) was thereafter assessed.

To study the shape of quasi-steady-state inactivation curve in peak I_{Na} taken with or without Gom A addition, the two-step voltage profile created though digital-to-analog conversion was employed. The relationships between the conditioning potentials (i.e., prepulses) and the normalized amplitude of peak I_{Na} were derived and

then least-squares fit by the nonlinear Boltzmann equation:

$$I = \frac{I_{max}}{1 + e^{\left\{\frac{(V - V_{\frac{1}{2}})qF}{RT}\right\}}}$$

where V is the conditioning potential (i.e., prepulse), $V_{1/2}$ the potential at which half-maximal inhibition occurs, I_{max} the maximal amplitude of peak I_{Na}, q the apparent gating charge in the inactivation curve (i.e., elementary charge [e] which occurs across the membrane electrical field between fully closed and open conformations), F the Faraday constant, R, the universal gas constant, T the absolute temperature, and RT/F = 25.2 mV.
